# Identifying New Frontiers for Social Media Engagement in Global Surgery: An Observational Study

**DOI:** 10.1007/s00268-020-05553-8

**Published:** 2020-05-23

**Authors:** Sergio M. Navarro, Dennis Mazingi, Evan Keil, Andile Dube, Connor Dedeker, Kelsey A. Stewart, Thando Ncube, Jennifer L. Rickard, Chris Lavy, Todd M. Tuttle

**Affiliations:** 1 Department of Surgery, University of Minnesota, Minneapolis, MN USA; 2 Nuffield Department of Orthopaedics, Rheumatology and Musculoskeletal Sciences, University of Oxford, Oxford, UK; 3 Department of Surgery, Parirenyatwa Group of Hospitals, Harare, Zimbabwe; 4 Department of Surgery, National University of Science and Technology, Bulawayo, Zimbabwe; 5 Department of Obstetrics and Gynecology, University of Minnesota, Minneapolis, MN USA; 6 University of the Witwatersrand, Linksfield Orthopaedic, Sports and Rehabilitation Centre, Johannesburg, South Africa

## Abstract

**Background:**

The purpose of this observational study is to characterize the use of social media content pertaining to global surgery.

**Methods:**

A search for public posts on social media related to global surgery was performed over a 3-month window, from January 1st, 2019, to March 31st, 2019. Two public domains were included in the search: Instagram and Twitter. Posts were selected by filtering for one hashtag: #GlobalSurgery. A binary scoring system was used for media format, perspective of the poster, timing of the post, tone, and post content. Data were analyzed using Chi-squared tests with significance set to *p* < 0.05.

**Results:**

Overall, 2633 posts with the hashtag #GlobalSurgery were publicly shared on these two social media platforms over the 3-month period. Of these, 2272 (86.3%) referenced content related to global surgery and were original posts. Physicians and other health professionals authored a majority (60.5%, 1083/1788) of posts on Twitter, whereas organizations comprised a majority of the posts on Instagram (59.9%, 290/484). Posts either had a positive (50.2%, 1140/2272) or neutral (49.6%, 1126/2272) tone, with only 0.3% or 6/2272 of posts being explicitly negative. The content of the posts varied, but most frequently (43.4%, 986/2272) focused on promoting communication and engagement within the community, followed by educational content (21.3%, 486/2272), advertisements (18.8%, 427/2272), and published research (13.2%, 299/2272). The majority of global surgery posts originated from the USA, UK, or Canada (67.6%, 1537/2272), followed by international organizations (11.5%, 261/2272). Chi-squared analysis comparing Instagram with Twitter performed examining media content, tone, perspective, and content, finding statistically significant differences (*p* < 0.001) the two platforms for each of the categories.

**Conclusion:**

The online social media community with respect to global surgery engagement is predominantly composed of surgeons and health care professionals, focused primarily on promoting dialogue within the online community. Social media platforms may provide a scalable tool that can augment engagement between global surgeons, with remaining opportunity to foster global collaboration, community engagement, education and awareness.

## Introduction

In recent years, the use of social media to disseminate information and promote global dialogue has grown. The total number of global social media users is estimated to grow to 3.29 billion users by 2022, representing 42.3% of the world’s population [1]. Social media continues to play a prominent role in promoting dialogue in particular with respect to global surgery. Several published studies have reported on the use of social media, including Instagram and Twitter, by patients, physicians, and hospitals in various fields, including orthopedics, transplant surgery, plastic surgery and neurosurgery [2–8].

To date, no such study to our knowledge exists for global surgery that characterizes the global landscape of how physicians, researchers, and collaborators are sharing their research and experiences. Dissemination of ideas and research in global surgery rely heavily on promoting dialogue, and awareness between researchers, institutions, and surgeons in the field-social media provides a framework for education, conversation, and increasing awareness. An ecosystem, prior to the advent of social media, was defined as a “functional unit with recognizable boundaries and an internal homogeneity” [9]. This definition allows an external analysis of a particular unit to be performed not only within biological systems, but also within virtual systems. Social media is a powerful tool for understanding interactions between those involved with global surgery and allows for a more detailed understanding of this rapidly growing ecosystem. Social media has seen increasing usage in lower- and middle-income countries (LMICs), with a reported 13% increase in social networking site usage from 2015 to 2017 according to Pew Research, indicating this tool ought to be utilized [10].

The purpose of this observational study was to analyze original publically shared global surgery content on social media platforms Instagram and Twitter in order to gain a deeper understanding of the interactions within social media with regard to global surgery. More specifically, we evaluated posts based on content related to global surgery for: (1) media format (picture or video); (2) tone (positive, negative, or neutral); (3) perspective; (4) content; (5) interactions (6) post popularity (number of likes); and (7) location of the author. We hypothesized that the global surgical community is diverse, inclusive, and representative of all nationalities, focused on education and expansion of global surgical efforts.

## Methods

A search for public posts on social media related to global surgery was performed over a 3-month window, from January 1st, 2019, to March 31st, 2019. Two public domains were included in the search: Instagram and Twitter. Posts were selected by filtering for one hashtag: #GlobalSurgery. Posts were selected by filtering for a single hashtag: #GlobalSurgery. Associated #GlobalSurgery hashtags were reviewed for relevance and potential inclusion, including #SoMeSurgery, #MedEdTwitter, #GlobalSurgeryDay, #GlobalPlasticSurgery, #GlobalCardiacSurgery, #TropicalMedicine, and #GlobalMedicine across both platforms, with #GlobalSurgery determined to be most specific and relevant to global surgery. A binary scoring system was used for media format, perspective of the poster, timing of the post, tone, and post content. Classification of perspective of the poster was assessed based on individual review of the tweet, poster handle and the poster's associated profile description. Data were analyzed using Chi-squared tests with significance set to *p* < 0.05.

### Inclusion criteria

All posts referencing global surgery were included, and those discussing other content were excluded. Only posts in English were included. Only posts relating to human participants were included, while veterinary and other nonhuman content was excluded.

Data were collected and analyzed by 2 independent reviewers (E.K. and C.D). Data were then reviewed by the first author (S.N.) Further interrater variability was resolved by review of original media and discussion to achieve agreement. No posts were excluded for interrater variability. Data analysis was performed in Microsoft Excel. Aggregate data totals as well as top poster and location details for the hashtag for Twitter were validated and confirmed using a third-party analytics software system (Symplur, LA, California, USA). Geographic location for individuals and institutions was determined by the author’s home country, as compared to the location of content contained within the individual post. For individuals and institutions posting content, posts were classified geographically based on the poster's country of origin rather than location of the post. For international NGOs posting content, posts were classified geographically as "international" rather than based on the country of origin of the international organization. Geospatial mapping of social media activity was conducted using a third-party geospatial mapping software system (ArcGis, San Francisco, California, USA) [11].

A binary categorical scoring system was used for media format, perspective of the author, tone, and content type. Posts were categorized by the primary type of media that was shared into three categories: text, picture, or video. Those determined to be “text” were the posts in which no picture or video was shared; thus, this did not occur on Instagram given the capabilities of the platform. If a post contained more than one media format, for example, both a photo and a video, the first item shared determined the sorted category. The perspective of the author was determined via analysis of the author's individual page and related posts. Tone was determined by overall explicit positive, negative, or neutral expression in the text accompanying any media based on a preset list of terms. Terms related to positive were “amazing,” “exciting,” and “great,” with accompanying exclamation points. Terms related to negative posts were “frustrating,” “inexcusable,” and “never.” All other posts were classified as neutral expressions.

Post visibility and popularity were determined by recording the number of hashtags and likes for each individual post, respectively. On Twitter, the number of replies and retweets were also recorded.

A temporal analysis of top posters and non-top posters was conducted, with qualitative assessment of drivers of activity completed. Posts from Twitter were classified by date and top 30% poster versus non-top 30% poster status.

## Results

**Fig. 1:**
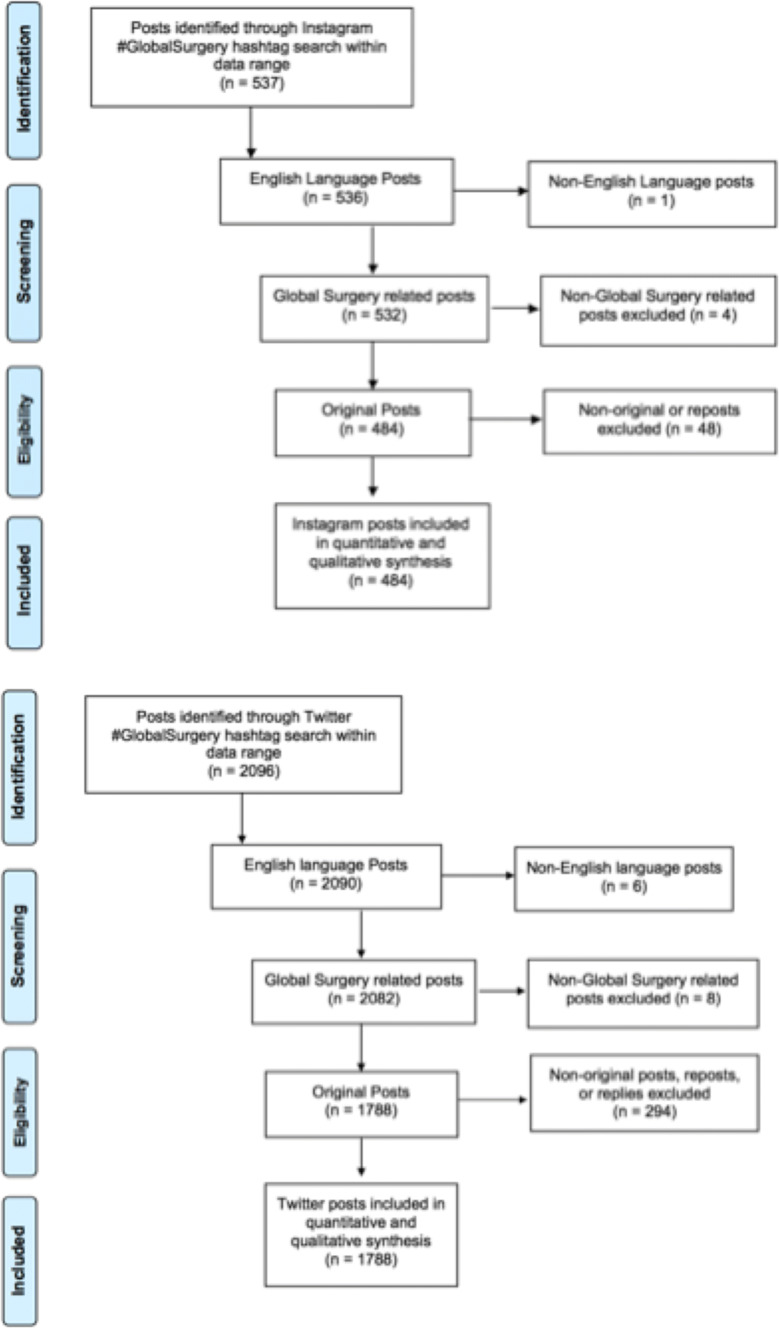
PRISMA based selection Process for #GlobalSurgery Instagram and Twitter Content

**Fig. 2:**
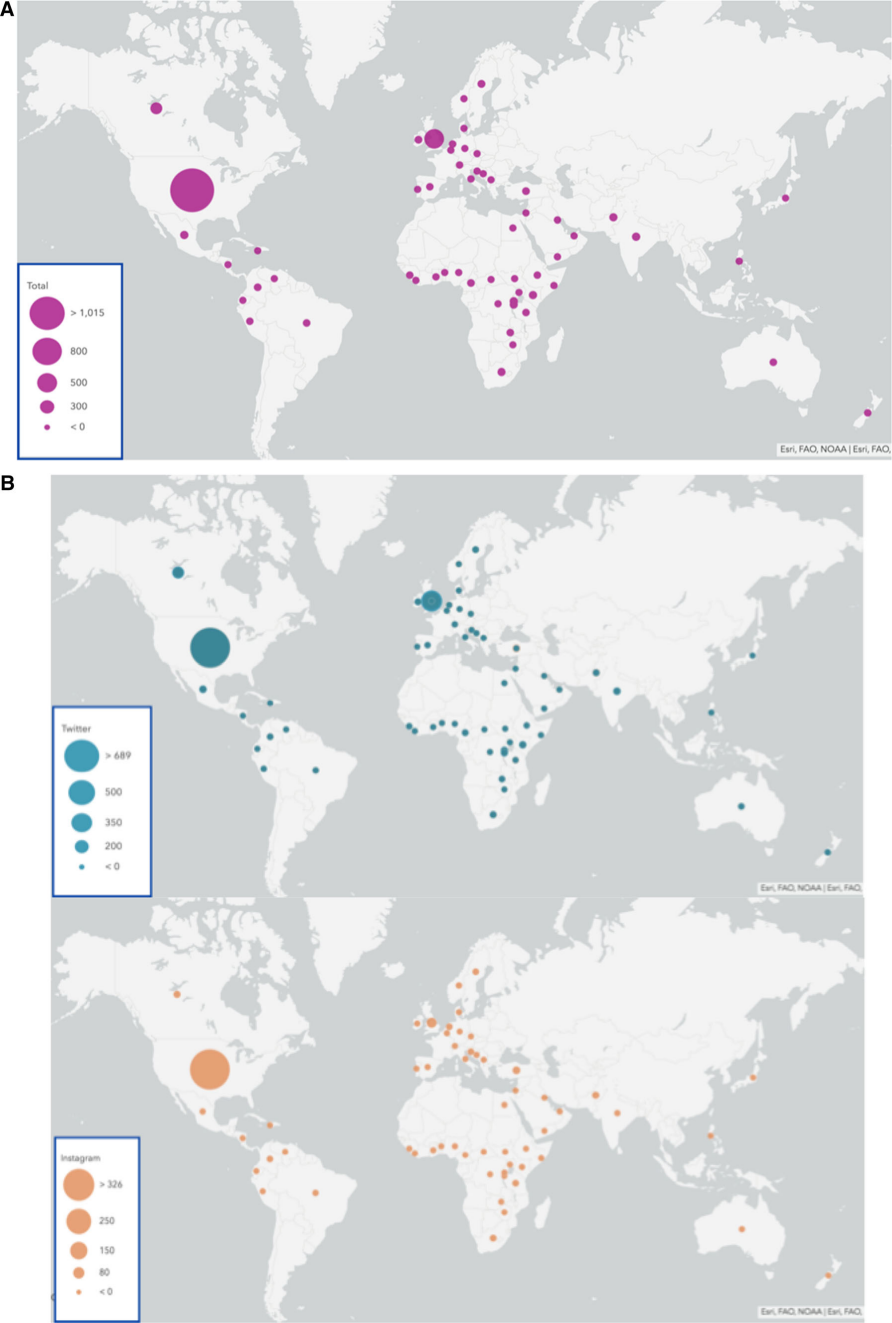
** A** Global Surgery social media activity from Jan 1, 2019 to Mar 30, 2019. **B** Global Surgery social media activity on Instagram and Twitter from Jan 1, 2019 to Mar 30, 2019

Overall, 2633 posts were reviewed, with 12 posts not associated with global surgery, 7 not in English, resulting in a total of 2614 posts related to Global Surgery with the hashtag #GlobalSurgery publicly shared on these two social media platforms over the 3-month period. Of these, 532 posts were reviewed from Instagram and 2082 from Twitter (Fig. 1). Of these, 2272 (86.3%) referenced content related to global surgery and were original posts. Physicians and other health professionals authored a majority (60.5%, 1083/1788) of posts on Twitter, whereas organizations comprised a majority of the posts on Instagram (59.9%, 290/484). Posts either had a positive (50.2%, 1140/2272) or neutral (49.6%, 1126/2272) tone, with only 0.3% or 6/2272 of posts being explicitly negative. The content of the posts varied, but most frequently (43.4%, 986/2272) focused on promoting communication and engagement within the community, followed by educational content (21.3%, 486/2272), advertisements (18.8%, 427/2272), and published research (13.2%, 299/2272). The majority of global surgery posts originated from the USA, UK, or Canada (67.6%, 1537/2272), followed by international organizations (11.5%, 261/2272), depicted in Fig. 2A. A full summary of results can be found in Table 1.

**Table 1:** Summary of #GlobalSurgery social media content by country

Dates: Jan 1st, 2019 to Mar 31st, 2019						
Country	Instagram (%)		Twitter (%)		Totals (%)	
USA	326	67.4	689	38.5	1015	44.7
UK	47	9.7	345	19.3	392	17.3
International organizations^1^	14	2.9	247	13.8	261	11.5
Canada	9	1.9	121	6.8	130	5.7
Mexico	2	0.4	29	1.6	31	1.4
India	2	0.4	29	1.6	31	1.4
Rwanda	1	0.2	28	1.6	29	1.3
Kenya	2	0.4	27	1.5	29	1.3
Pakistan	12	2.5	16	0.9	28	1.2
Burundi	0	0.0	25	1.4	25	1.1
South Africa	7	1.4	17	1.0	24	1.1
Sweden	5	1.0	15	0.8	20	0.9
Africa	0	0.0	18	1.0	18	0.8
Colombia	4	0.8	12	0.7	16	0.7
Turkey	15	3.1	1	0.1	16	0.7
Others^2,3,4^	38	7.9	169	9.4	207	9.1

^1^International organizations included InciSioN, ReSurge, the G4 Alliance, ISS SIC, Women Surgeons, and Global Surgery Students

^2^Others for Instagram include Tanzania 6, Netherlands 5, Croatia 5, Brazil 3, Kuwait 3, Norway 3, Unable to determine 2, Australia 1, Spain 1, Italy 1, Ghana 1, Somalia 1, Liberia 1, Democratic Republic of the Congo 1, Nigeria 1, United Arab Emirates 1, Bolivia 1, France 1

^3^Others for Twitter include Cameroon 15, Zambia 11, Australia 10, Brazil 9, Ethiopia 9, Sierra Leone 9, New Zealand 8, Peru 8, Spain 7, Europe 6, Italy 5, Switzerland 5, Tanzania 4, Ghana 4, Somalia 4, Yemen 4, Philippines 4, Netherlands 3, Liberia 3, United Kingdom 3, Egypt 3, Democratic Republic of the Congo 2, Nicaragua 2, Belgium 2, Venezuela 2, Bosnia and Herzegovina 2, Czech Republic 2, Denmark 2, Ecuador 2, Kosovo 2, South Sudan 2, Uganda 2, Croatia 1, Nigeria 1, United Arab Emirates 1, Israel 1, Philippines 1, Benin 1, East and Central Africa 1, Germany 1, Haiti 1, Japan 1, Portugal 1, West Africa 1, Zimbabwe 1

^4^Others for Totals include Cameroon 15, Brazil 12, Zambia 11, Australia 11, Tanzania 10, Ethiopia 9, Sierra Leone 9, New Zealand 8, Peru 8, Spain 8, Netherlands 8, Italy 6, Europe 6, Croatia 6, Switzerland 5, Ghana 5, Somalia 5, Yemen 4, Philippines 4, Liberia 4, United Kingdom 3, Democratic Republic of the Congo 3, Egypt 3, Kuwait 3, Norway 3, Nicaragua 2, Nigeria 2, Belgium 2, Venezuela 2, Bosnia and Herzegovina 2, Czech Republic 2, Denmark 2, Ecuador 2, Kosovo 2, South Sudan 2, Uganda 2, United Arab Emirates 2, Unable to determine 2, Israel 1, Philippines 1, Benin 1, East and Central Africa 1, Germany 1, Haiti 1, Japan 1, Portugal 1, West Africa 1, Zimbabwe 1, Bolivia 1, France 1, Brazil 1

### Instagram

On Instagram, 537 posts with the hashtag #GlobalSurgery were publicly shared over the 3-month period. Of these, 484 (86.3%) referenced content related to global surgery and were original posts. Organizations comprised a majority of the posts on Instagram (59.9%, 290/484). Posts were largely positive (76.2%, 368/484) or neutral (23.6%, 114/484) tone, with only 1 post noted as being explicitly negative. The content of the posts varied, but most frequently (51.2%, 248/484) focused on promoting communication and engagement within the community, followed by advertisements (18.6%, 90/484) and educational content (14.3%, 69/484). The mean number of likes per post was 488.8, representing the popularity of the posts. Hashtags used per post averaged 11.4 hashtags per post on Instagram. The majority of global surgery posts originated from the USA, UK, and international organizations (80.0%, 387/484), depicted in Fig. 2B. Of LMICs, Pakistan (2.5%, 12/484) was the top country represented.

### Twitter

Overall, 2,096 posts with the hashtag #GlobalSurgery were publicly shared on Twitter over the 3-month period. Of these, 1788 (85.2%) referenced content related to global surgery and were original posts. Physicians and other health professionals authored a majority (60.5%, 1083/788) of posts on Twitter. Posts either had a positive (43.1%, 771/1,788) or neutral (56.6%, 1126/1788) tone, with only 0.3% or 5/1,788 of posts being explicitly negative. The content of the posts varied, but most frequently (41.3%, 771/1788) focused on promoting communication and engagement within the community, followed by educational content (23.3%, 417/1788), advertisements (18.8%, 337/1788), and published research (15.7%, 281/1788). Posts averaged nearly 10 likes/post, with 4.33 retweets/post, and 0.36 replies/post, representing the popularity of the posts. Hashtags used per post averaged 2.8 hashtags per post on Twitter. The majority of global surgery posts originated from the USA, UK, or Canada (64.6%, 1155/1619), followed by international organizations (13.8%, 247/1619), depicted in Fig. 2B. Of LMICs, Rwanda (1.6%, 28/1619), Kenya (1.5%, 27/1619), and Burundi (1.4%, 25/1619) made up the top countries represented.

### Comparison

Chi-squared analysis performed comparing Instagram with Twitter for media content, tone, perspective and content found statistically significant differences between the two (*p* < 0.001). This comparison is visualized in Table 2. Post visibility and popularity between the global surgery content between the two platforms was unable to be compared.

**Table 2:** Summary of Social Media Content

Summary of #GlobalSurgery Social Media Content					
Dates: Jan 1st, 2019 to Mar 31st, 2019					
Categories	Instagram (%)		Twitter (%)		*p*-value
Media format					
Picture	458	94.6	768	43.0	< 0.001
Video	26	5.4	36	2.0	
Text	0	0.0	984	55.0	
Tone					
Positive	369	76.2	771	43.1	< 0.001
Neutral	114	23.6	1012	56.6	
Negative	1	0.2	5	0.3	
Perspective					
Patient	2	0.4	0	0.0	< 0.001
Physician/individual	135	27.9	1083	60.6	
Friend/family	1	0.2	2	0.1	
Business	56	11.6	128	7.2	
Professional organization	290	59.9	575	32.2	
Content					
Education	69	14.3	417	23.3	< 0.001
Advertisement	90	18.6	337	18.8	
Public image	248	51.2	738	41.3	
Research	18	3.7	281	15.7	
News	0	0.0	3	0.2	
Personal experience	59	12.2	12	0.7	
Visibility					
#hashtags/post	11.4		2.8		
Popularity					
Likes/post	488.8		9.7		
Retweets/post			4.3		
Replies/post			0.4		

### Top poster analysis

**Fig. 3:**
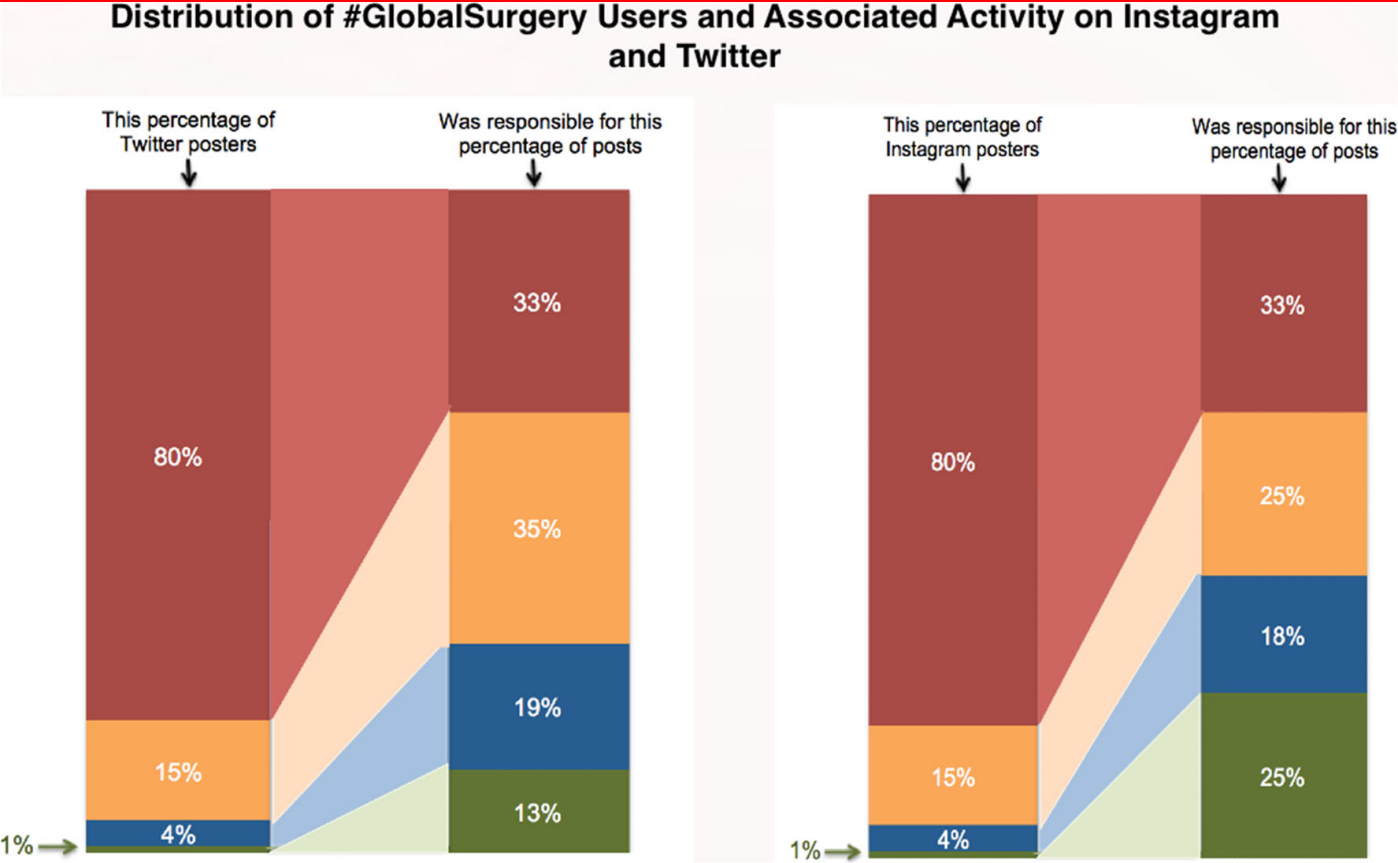
Distribution of Global Surgery Social media users and associated activity on Instagram and Twitter from Jan 1, 2019 to Mar 30, 2019

To determine whether an equitable conversation of social media users made up the global surgery community, with a generally equal distribution of tweets among all users, we analyzed the ratio of top tweeters to the bottom percentile of #GlobalSurgery Twitter users. Analysis of the top percentage of posters related to their associated content on Instagram and Twitter is available in Fig. 3. The analysis determined that the top 20th percentile #GlobalSurgery posters on Instagram comprised nearly 67% of overall global surgery content (324/484), driven primarily by MercyShips (29.1%, 141/484). The top 20 #GlobalSurgery posters on Twitter comprised approximately 30% of overall #GlobalSurgery Twitter Content, driven by the top 10 posters (21.1%, 378/1619). We analyzed the number of tweets published by the top 20% most prolific tweeters as a ratio of the number of tweets published by the bottom 20% tweeters, analogous to the 20/20 ratio, or the Quintile Share Ratio ("QSR"), used for determination of income inequality [12]. The determined ratio of tweets by top 20% tweeters to bottom 20% tweeters was 25:1 (*n* = 650/25).

### Temporal analysis

**Fig. 4:**
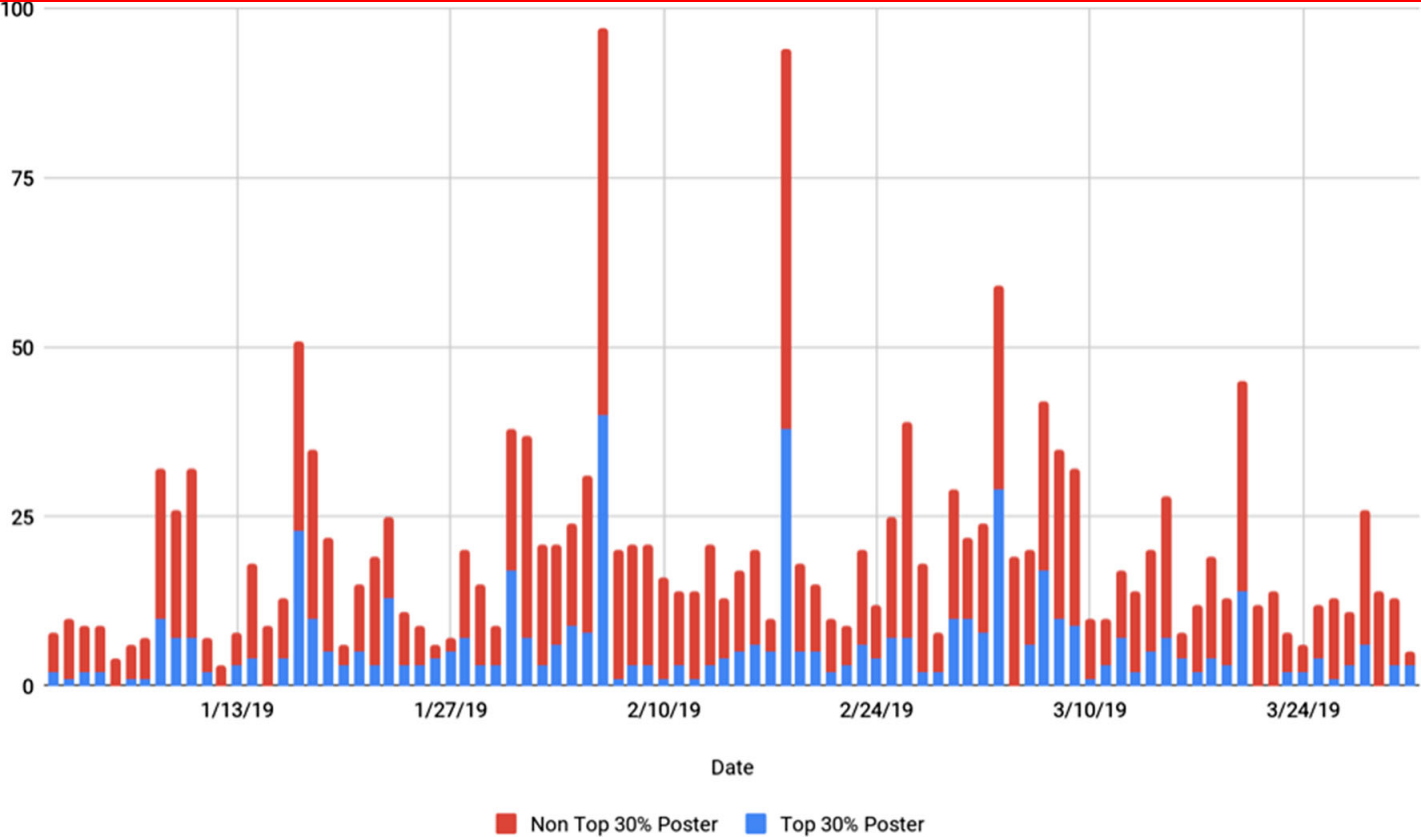
Temporal analysis of #GlobalSurgery Twitter activity from Jan 1, 2019 to Mar 30, 2019

Secondary analysis of the temporal distribution of content found noted variation in terms of number of tweets per day, in addition to the number of different geographic locations generated over the course of the study. #GlobalSurgery Twitter content averaged 19.9 posts/day (± 15.8 posts/day), with steady-state content generated by the Top 30% posters averaging 5.7 posts/day (± 6.94 posts/day) and non-Top 30% posters averaging 14.1 posts/day (± 9.7 posts/day). A summary of the analysis is available in Fig. 3. The influence of two tweet-based global surgery journal clubs on the volume of posts is clear on 2/6/19 and 2/19/19, further stressing the role that top posters play in the discussion around global surgery [13]. Of note, there were three captured monthly online Twitter Journal Club sessions specifically led and created regarding global surgery, leading to increased participation and activity within the global surgery social media community. Secondary increases in activity were identified corresponding to noted global surgery conferences, hosted by the Academic Surgical Conference and Karunya Institute of Technology and Sciences (KITS) International Symposium on Surgical Innovations and Healthcare and Hackathon, as well as corresponding to a special edition journal release regarding global surgery by the British Journal of Surgery occurring during the period of the study (Fig. 4) [14].

## Discussion

Global surgery is a burgeoning interdisciplinary field of global health that aims to provide improved and equitable surgical care in less well served places around the globe [15]. While the impetus for much of the work in global surgery originates in high-income countries, participation by stakeholders in the destination countries **is** considered crucial to the success of global surgery work. It is a necessarily collaborative endeavor involving exchange of expertise, funding, data and research between stakeholders in HIC’s and LIC’s [16, 17]. To be effective, partnerships in global surgery should be a bidirectional ‘conversation’ between the two groups. With social media an available tool able to connect distant parts of the world nearly instantaneously, as well as enable conversations between health care professionals and the general public, platforms such as Twitter and Instagram enable education, dialogue, and partnerships in ways never before. This study enables an initial look at how well information disseminated via social media platforms about global surgery are fulfilling these desirable goals of enabling education, conversation, and partnerships. Given that English is widely considered the language of medicine and posts in English were made in over 70 countries, only posts in English were included [18]. A total of 7 posts were excluded for being in a language other than English.

### Geographic distribution

The geographic distribution of social media content generated suggests that the content generated in global surgery social media conversation is predominantly originating in the global north, and dominated by health care professionals and official organizations, as seen in Fig. 2A , B. 70% of all social media communication about global surgery analyzed originated in the USA and the UK. The observed disparity may be explained by lower social media usage in LMICs. One might presume that this could be driven by reduced usage of social media in the global south, but studies indicate that social media usage is quite high and mobile penetration is generally high across Africa and LMICs [10, 19]. The geographic distribution of tweets and impressions is important, as global health space endeavors should bridge beyond conversations between a smaller academic ecosystem by seeking to engage practitioners and educating patients in LMICs in a meaningful way. While social media usage is quite common in LICs, this could suggest that there may be questions regarding the specific role and impact of social media by regional users with respect to participating in the global surgery community. This may outline an increasing opportunity for surgeons from LMICs to see a role to drive ‘potential impact’ by using social media to promote the global surgery agenda. Another plausible explanation for the seemingly limited geographic distribution could be the “echo chamber effect.” This may describe a lack of divergent viewpoints and diversity in participants in social media conversations because of personal or institutional biases reinforced by self-curation [20].

### Influential posters

In terms of the most influential posters, we found that the top ten tweeters accounted for a significant percentage of total tweets (20% of the entire population), with the top twenty tweeters comprising 30% of total activity. The top two Twitter influencers identified in the study alone accounted for nearly 5% of all tweets analyzed. The top 20% of Twitter and Instagram posters were responsible for 67% of posts, respectively, as seen in Fig. 3. This signifies that a smaller group of people and organizations alone drive and comprise a large percentage of the total number of tweets. Such social media influencers are responsible for leading and driving global surgery conversation, but may not represent a diverse subset of the ideas and opinions disseminated within the global surgical community. This is supported by a 2009 Harvard Business Review study showing that 10 percent of users sent about 90 percent of all tweets as well as a more recent 2019 Pew Research Centre study that showed similar findings [21, 22].

### Temporal analysis

In terms of activity, temporal analysis showed a relatively small group of users talking to each other for extended intervals, joined by a broadening group of individuals and organizations at specific intervals. That said, during specific periods, there was general increased note growth in social media posts at specific time intervals, as well as increases noted specifically during conferences and Twitter chats. This reinforces the utility of online social media journal clubs in engaging users in addition to the use of social media during conferences to widen participation in global academic activities.

### Limitations

There were several limitations of this study, as any posts not including the hashtag #GlobalSurgery related to global surgery were not included. This means that much of the content not in the English language and those not with #GlobalSurgery related hashtags but yet related to global surgery were not included in this analysis. Furthermore, hashtags are an effective way to sort through the millions of posts and tweets from Instagram and Twitter each day [23–25]. Market research has shown that Tweets with one or more hashtag are 55 percent more likely to be retweeted [26].The hashtag #GlobalSurgery was determined to give the most representative look within global surgery efforts across the globe. This was done after analyzing the prevalence of other hashtags such as #GlobalSurgeryDay, #GlobalPlasticSurgery, #GlobalCardiacSurgery, #TropicalMedicine, and #GlobalMedicine across both platforms. No other hashtags gave a more detailed look at global surgery efforts and had a high enough prevalence to allow for a representative sample within a three month time period. For example, #SoMESurg is a recently popular hashtag notable to trend on Twitter and other social media platforms, although non-specific to global surgery. Social media data do not necessarily approximate the global surgery activity. Social media content was private and non-public and thus excluded from this analysis. Furthermore, we analyzed the country of the social media account rather than the country in which the social media post originated, a potential oversimplification of the country of representation. Finally, social media changes over time and is not permanent, and a longer time period of analysis would have helped capture more longer term trends, especially temporal trends. Of note, two global surgery conferences occurred within the time period that contributed to noted increases in social media posting, namely the Academic Surgical Congress conference, held from February 5, 2019, to February 7, 2019, as well as the Karunya Institute of Technology and Sciences (KITS) International Symposium on Surgical Innovations and Healthcare and Hackathon, held at KITS in conjunction with the Harvard Program in Global Surgery and Social Change (PGSSC) from February 18, 2019, to February 20, 2019.

### Promoting global surgery engagement

As global surgeons, we would encourage brainstorming and proposing ways to ensure dialogue is inclusive and truly global and representative in terms of LMIC equality. Global surgery groups such as Mercy Ships have shown their effectiveness at promoting engagement and awareness of their mission. SurgAfrica has accordingly united global surgeons, in online platforms in WhatsApp to develop a tool to stimulate learning, facilitate decision making, and promote discussions by the future of the operating room as well as other studies have explored usage in other clinical settings [27, 28]. Continued use of social media platforms as an opportunity and a tool to involve global surgeons and medical students worldwide could be used to disseminate research, foster partnerships, promote collaboration and awareness while refrain from self-promotion. Social media communities and networks usage by the global surgery community should be welcomed and embraced, changing the field for the better.

## Conclusion

The online social media community with respect to global surgery engagement is predominantly composed of surgeons and health care professionals, focused primarily on promoting dialogue within the online community. Social media platforms provide a scalable tool that can augment engagement between global surgeons, with remaining opportunity to foster global collaboration, community engagement, education and awareness [28–30]. While the global surgical community is diverse, inclusive, and representative of all nationalities, the current social media landscape shows a less diverse, inclusive view of global surgery. Efforts encouraging a more inclusive platform as well as a more globally inclusive audience are needed, as well as education of social media usage. The creation and emergency of a formal Global Surgery Social Media Network, similar to the Thoracic Surgery Social Media Network, with established #SoMe leadership by country, inclusive of academic institutions, surgeons, resident physicians, and medical students as partners, may help further representation and promote dialogue [31, 32]. With increased focus on education and expansion of global surgical efforts across social media platforms, there is significant opportunity to ensure equality and diversity of views shared and expressed globally.

## References

[1] Appel G, Grewal L, Hadi R, Stephen AT (2020). The future of social media in marketing. J Acad Mark Sci.

[2] Alotaibi NM, Badhiwala JH, Nassiri F (2016). The current use of social media in neurosurgery. World Neurosurg.

[3] Sorice SC, Li AY, Gilstrap J (2017). Social media and the plastic surgery patient. Plast Reconstr Surg.

[4] Henderson ML, Adler JT, Van Pilsum Rasmussen SE (2019). How should social media be used in transplantation? A survey of the american society of transplant surgeon. Transplantation.

[5] Haeberle HS, Egger AC, Navarro SM (2017). Social media and pediatric scoliosis: an analysis of patient and surgeon use. Surg Technol Int.

[6] Ramkumar PN, Navarro SM, Cornaghie MM (2018). Social media in shoulder & elbow surgery: an analysis of twitter and Instagram. Int J Sports Med.

[7] Ramkumar PN, Navarro SM, Haeberle HS (2017). Social media and total joint arthroplasty: an analysis of patient utilization on Instagram. J Arthroplasty.

[8] Navarro SM, Haeberle HS, Cornaghie MM, et al (2017) The impact of social media in medicine: an examination of orthopaedic surgery. In: Ahern T (ed) Social media: practices, uses, and global impact. Nova Science, pp 155–172

[9] Scheffer M, Brock W, Westley F (2000). Socioeconomic mechanisms preventing optimum use of ecosystem services: an interdisciplinary theoretical analysis. Ecosystems.

[10] Pew Research Center (2018). Social media use continues to rise in developing countries but plateaus across developed ones.

[11] ESRI (2013) ArcGIS desktop: release 10.2. Redlands CA

[12] Drezner T, Drezner Z, Hulliger B (2014). The Quintile Share Ratio in location analysis. Eur J Oper Res.

[13] NihIci T, Archer M, Harrington C (2020). Trainee thoracic surgery social media network: early experience with tweetchat-based journal clubs. Ann Thoracic Surg.

[14] Bolton WS, Aruparayil N, Quyn A (2019). Disseminating technology in global surgery Br. Br J Surg.

[15] Dare AJ, Grimes CE, Gillies R (2014). Global surgery: defining an emerging global health field. Lancet.

[16] Azzie G, Bickler S, Farmer D, Beasley S (2008). Partnerships for developing pediatric surgical care in low-income countries. J Pediatric Surg.

[17] Riviello R, Ozgediz D, Hsia RY (2010). Role of collaborative academic partnerships in surgical training, education, and provision. World J Surg.

[18] Baethge C (2008). The Languages of medicine. Dtsch Aerzteblatt Online.

[19] Hagg E, Dahinten VS, Currie LM (2018). The emerging use of social media for health-related purposes in low and middle-income countries: a scoping review. Int J Med Inform.

[20] Duseja N, Jhamtani H (2019) A sociolinguistic study of online echo chambers on twitter. In: Proceedings of the third workshop on natural language processing and computational social science. 10.18653/v1/W19-2109

[21] Pew Research Center (2019). Sizing up twitter users.

[22] Heil B, Piskorski M (2009) New twitter research: men follow men and nobody tweets. Harvard Business Review.

[23] Gruzd A, Haythornthwaite C (2013). Enabling community through social media. J Med Internet Res.

[24] Cunha E, Magno G, Comarela G, et al (2011) Analyzing the dynamic evolution of hashtags on twitter: a language-based approach. In: LSM '11 Proceedings of the workshop on languages in social media, pp 58–65

[25] Twitter (2018) How to use hashtags. In: Twitter Help Cent.

[26] Himelboim I, Smith MA, Rainie L (2017). Classifying Twitter topic-networks using social network analysis. Soc Media Soc.

[27] Chan WSY, Leung AYM (2018). Use of social network sites for communication among health professionals: systematic review. J Med Internet Res.

[28] Pittalis C, Brugha R, Crispino G (2019). Evaluation of a surgical supervision model in three African countries—protocol for a prospective mixed-methods controlled pilot trial. Pilot Feasibility Stud.

[29] Vohra RS, Hallissey MT (2015). Social networks, social media, and innovating surgical education. JAMA Surg.

[30] Topf JM, Hiremath S (2015). Social media, medicine and the modern journal club. Int Rev Psychiatry.

[31] Luc JGY, Ouzounian M, Bender EM (2019). The thoracic surgery social media network: early experience and lessons learned. J Thorac Cardiovasc Surg.

[32] Antonoff MB (2015). Thoracic surgery social media network: bringing thoracic surgery scholarship to twitter. J Thorac Cardiovasc Surg.

